# Inborn errors of immunity affecting the MHC class I pathway for antigen presentation

**DOI:** 10.70962/jhi.20250029

**Published:** 2025-06-11

**Authors:** Stephan D. Gadola, Ömür Ardeniz, Angelica Cuapio, Jacques Zimmer, Hans-Gustaf Ljunggren

**Affiliations:** 1 Department of Rheumatology and Pain Medicine, Bethesda Hospital, Basel, Switzerland; 2 Department of Immunology, Faculty of Medicine, Ege University, Izmir, Turkey; 3 Center for Infectious Medicine, Department of Medicine Huddinge, Karolinska Institutet, Stockholm, Sweden; 4 Department of Infection and Immunity, Luxembourg Institute of Health, Esch-sur-Alzette, Luxembourg

## Abstract

Inborn errors of immunity affecting the major histocompatibility complex (MHC) class I pathway for antigen presentation represent a rare group of human disease syndromes. Here, we review symptoms associated with such conditions, which manifest as chronic respiratory infections, granulomatous skin lesions, and related severe symptoms. We highlight the potential for misdiagnosis with autoimmune conditions such as granulomatosis with polyangiitis and emphasize the necessity of infection-focused treatment, given the risks associated with immunosuppressive therapy. Furthermore, we present novel long-term follow-up data on TAP-deficient patients, revealing new insights into disease progression, including an increased risk of skin cancer and severe herpesvirus infections. Additionally, we discuss cases of a few individuals with significant MHC class I deficiency who remain largely asymptomatic, underscoring the variability in clinical presentation. Our findings emphasize the importance of further genetic research and immunopathological analysis to identify predictive markers and optimize individualized treatment approaches. Long-term patient surveillance remains critical to understanding late-onset complications and refining clinical management strategies.

## Teaser

The paper discusses rare inborn errors of immunity affecting MHC class I antigen processing, leading to infections and skin lesions, often misdiagnosed as autoimmune disease. It emphasizes the need for infection-focused care, highlights disease variability, and presents long-term data to guide personalized treatment.

## Introduction

The immune system constitutes an intricate network of cells and soluble factors, crucial for defending the body against a wide range of pathogenic threats including viruses, bacteria, parasites, and fungi, as well as malignant cells. Central to this defense is the process of antigen recognition, which enables immune cells to recognize and distinguish most often harmless “self” antigens from potentially harmful “non-self” antigens.

The major histocompatibility complex (MHC) class I pathway for antigen processing plays a pivotal role in this process by continuously displaying fractions of degraded intracellular proteins on cell surface–bound MHC class I molecules (1, 2, 3, 4). The latter are continuously scrutinized by cytotoxic CD8^+^ T cells (5). In parallel, natural killer (NK) cells (6) monitor cells that have lost the expression of MHC class I molecules (7, 8), the latter referred to as “missing self” recognition (9). Together, these two systems cooperate in a surveillance mechanism that continuously serves to detect and eliminate aberrant cells in the normal body.

Importantly, the expression of MHC class I molecules is also essential for the development of CD8^+^ T cells (10) and for the maintenance of full functionality of NK cells (11, 12, 13). In the absence of MHC class I molecules, the selection of CD8^+^ T cells in the thymus is largely impaired, resulting in low numbers of naïve CD8^+^ T cells in the periphery (14, 15, 16). In contrast, NK cells undergo normal development in the absence of MHC class I molecules but become functionally impaired and lose the ability to recognize the absence of self-MHC class I (11, 12, 13, 17, 18).

## Inborn errors of immunity (IEI)

IEI are a large group of genetically inherited disorders that affect various components of the immune system (19, 20). These disorders result from mutations in genes essential for immune cell development, function, or signaling pathways, leading to immune dysregulation. They include, but are not limited to, increased susceptibility to infections, inflammatory processes, lymphoproliferative disorders, autoimmune diseases, or predispositions for various malignant diseases. In recent decades, research into IEI has not only deepened our understanding of associated conditions but has also, importantly, provided deeper insights into immunological processes and function of the human immune system (19, 20).

## IEI affecting the MHC class I expression

**Figure 1.**
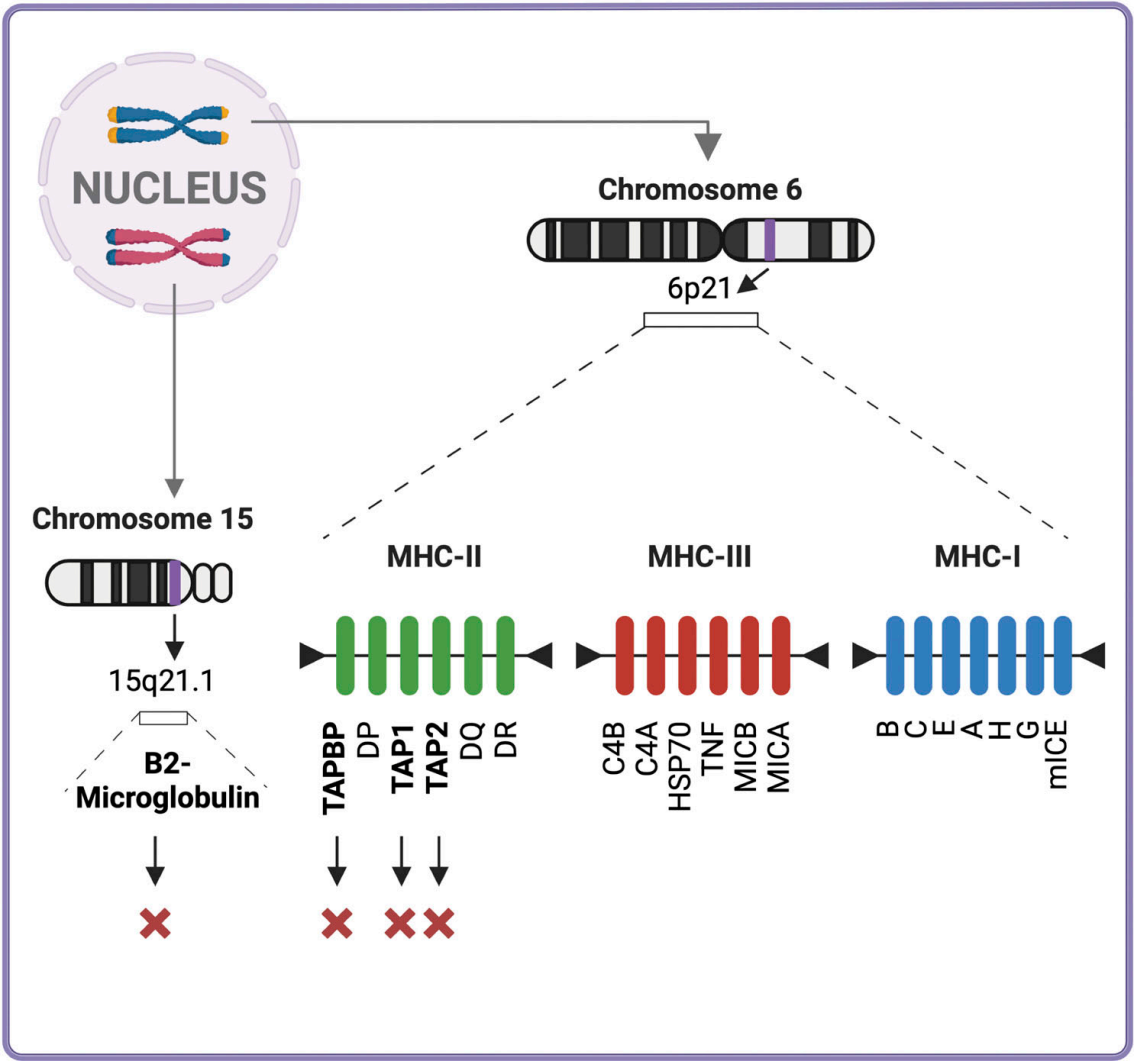
**Genetic map of the MHC region.** Overview of the human MHC, also known as the HLA system. Located on chromosome 6p21.3, the MHC is a densely packed, highly polymorphic genomic region encompassing >200 genes. Among these are the classical class I and class II loci, which encode cell surface proteins crucial for antigen processing, presentation, and immune recognition by T cells and NK cells. The figure is simplified to highlight the spatial organization of key genes within the MHC, including those that encode proteins essential for MHC class I–mediated antigen presentation, such as the TAP peptide transporters and the peptide-loading complex component tapasin. Their close genomic proximity suggests coordinated regulation, reflecting evolutionary pressures to maintain robust antigen presentation and overall immune homeostasis. Additionally, the figure indicates the location of the β_2_m gene on chromosome 15q21.1, which is vital for proper MHC class I assembly and expression. Genes discussed in the present review associated with loss of MHC class I expression are marked with a cross (X).

One category of IEI, previously referred to as bare lymphocyte syndrome type 1, affects MHC class I expression (21, 22, 23). According to the most recent international update on human IEI, MHC class I deficiency can result from mutations in the genes encoding the transporter associated with antigen processing 1 and/or 2 (*TAP1 and/or 2*) as well as genes encoding the TAP-binding protein/tapasin (*TAPBP*) and β_2_-microglobulin (*β*_*2*_*m*) (24, 25) (Fig. 1). Together, these conditions represent an ultra-rare category of IEI (22, 23, 26), with fewer than 50–100 cases of MHC class I deficiencies (all genetic causes combined) documented. Publicly available population‐genetics resources (e.g., gnomAD, Human Gene Mutation Database, and ClinVar) confirm that true loss‐of‐function (LOF) alleles in *TAP1*, *TAP2*, *TAPBP*, or *β*_*2*_*m*, especially in the homozygous state, are exceedingly rare. Consequently, diseases caused by biallelic LOF mutations in these genes are extremely uncommon, with an estimated prevalence on the order of <1 per 1,000,000. No larger population study has provided new, more precise prevalence figures. Thus, most sources continue to describe these disorders in terms of case reports rather than broad epidemiological data.

In this article, we present new insights into the clinical manifestations of these very unusual disorders, focusing primarily on defects in the *TAP1* and *TAP2* genes, since these are relatively more frequent than those affecting *β*_*2*_*m* or *TAPBP* genes. However, before examining these conditions, we first review the principles of MHC class I–mediated antigen processing and presentation. A solid grasp of this mechanism is essential for understanding how disruptions in any component of the pathway can prevent stable peptide loading onto MHC class I molecules, and, consequently, their expression at the cell surface.

## MHC class I pathway for antigen processing and presentation

**Figure 2.**
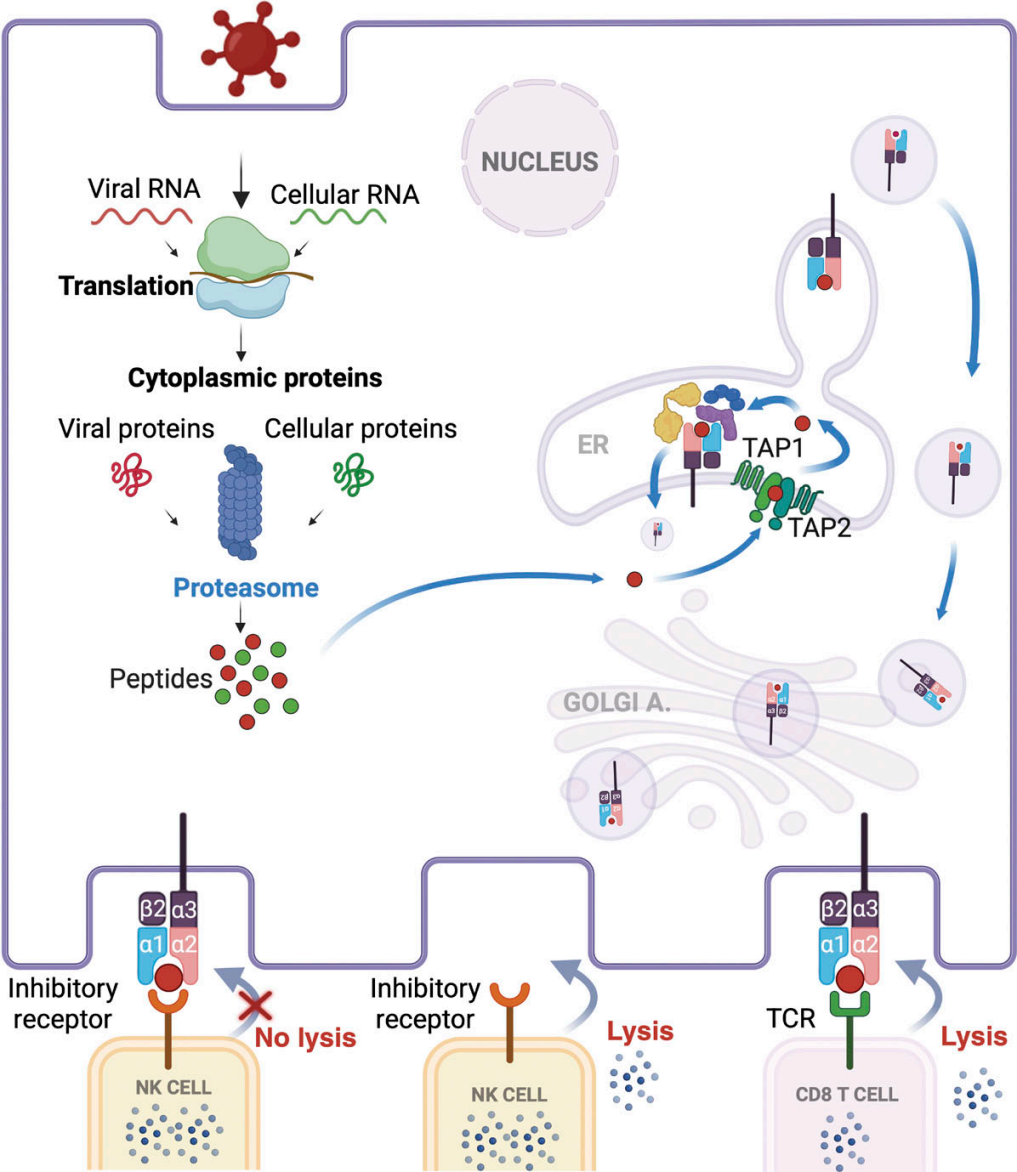
**MHC class I–mediated antigen processing and presentation.** Illustration of the cellular mechanisms governing the processing and presentation of antigens on MHC class I molecules, a pathway essential for immune surveillance against intracellular pathogens and malignancies. The process begins with the translation of RNA (which could be both of endogenous cellular or viral origin) into proteins within the cytoplasm. A subset of these newly synthesized proteins is degraded by the proteasome into peptide fragments, often 8–16 amino acids in length. Under inflammatory conditions, a specialized immunoproteasome can be induced, further optimizing peptide generation for MHC class I loading. The transporter associated with antigen processing (TAP) complex then translocates the peptides from the cytosol into the ER. Once inside the ER, peptides are trimmed by ER aminopeptidases to achieve optimal length (usually 8–10 amino acids) for binding MHC class I molecules. Assembly of the MHC class I heavy chain and β_2_m with these peptides is facilitated by the peptide-loading complex (PLC), comprising chaperones such as tapasin, ERp57, and calreticulin. Upon successful peptide binding, the stable MHC class I–peptide complex dissociates from the PLC and is transported to the cell surface via the secretory pathway. At the plasma membrane, peptide–MHC class I complexes are surveyed primarily by CD8^+^ T cells and NK cells. CD8^+^ T cells use their TCR to recognize specific peptide–MHC class I complexes and can eliminate infected or transformed cells. In contrast, NK cells detect the presence of MHC class I via inhibitory receptors (e.g., killer cell immunoglobulin-like receptors or NKG2A), thereby sparing healthy cells. When MHC class I expression is reduced or lost—often during viral infection or tumor progression—NK cells are released from inhibition and can eliminate the aberrant cell. Collectively, the figure underscores the critical role of MHC class I antigen presentation in coordinating innate and adaptive immunity, enabling effective defense against a broad range of intracellular threats.

The MHC class I pathway for antigen processing and presentation is responsible for displaying peptides derived from intracellular proteins on the surface of all nucleated cells (Fig. 2). This allows the immune system to constantly survey patterns of the host proteome and its potential modifications. The MHC class I antigen processing and presentation pathway is well characterized (3, 4). Briefly, the process begins with the degradation of samples of intracellular proteins in the proteasome, a proteolytic complex that breaks down targeted proteins into smaller peptide fragments (56). These peptides are then transported from the cytoplasm into the endoplasmic reticulum (ER). The translocation of peptides into the ER is mediated by the TAP peptide transporters, heterodimeric complexes composed of TAP1 and TAP2 subunits (57, 58). Once inside the ER, peptides are trimmed to an optimal length (usually 8–10 amino acids) and are subsequently loaded onto the MHC class I molecules in complex with β_2_m (3). Here, chaperone proteins including tapasin, calreticulin, and ERp57, ensure proper folding and loading of the peptides (59, 60). After peptide loading, the MHC class I molecules exit the ER via the Golgi apparatus and are transported to the cell surface (3).

The integrity and efficiency of the MHC class I pathway depend heavily on the proper functioning of the TAP, tapasin, and β_2_m proteins (61); consequently, LOF mutations in genes encoding these proteins severely impair antigen presentation on MHC class I molecules. For example, without the TAP-mediated transport of peptides into the ER, MHC class I molecules cannot be properly loaded, collapse, and consequently fail to reach the cell surface (3, 4, 62). The immunological consequences of such a defect have been characterized in detail in many experimental model systems (13, 14, 15, 16, 63). In the sections that follow, we examine the pathological manifestations observed in TAP-deficient patients and outline key considerations for their clinical management. We also provide a brief overview of other known IEI related to MHC class I, including defects in β_2_m and tapasin.

## Human TAP1 and TAP2 deficiencies

Human TAP deficiencies are caused by mutations in the genes encoding TAP1 and/or TAP2. The condition is characterized by a significant reduction of MHC class I molecules on the surface of nucleated cells. While TAP1 and/or TAP2 deficiency can manifest with a wide spectrum of symptoms, respiratory tract infections and autoinflammatory granulomatous skin lesions are by far the most common clinical manifestations (22, 23, 31, 32, 33, 37, 39, 41, 50, 64). TAP deficiency follows an autosomal recessive pattern, and parents of affected individuals are often consanguineous (e.g., uncle-niece [34] or first cousins [37, 65]). In rare instances, the parents may be more distantly related, such as third cousins (40), or appear not to be consanguineous at all (42). The latter scenario can arise when both parents share a common human leukocyte antigen (HLA) haplotype harboring the same *TAP* gene mutation (22). Because most reported TAP-deficient patients come from consanguineous backgrounds, leading frequently to universal homozygosity across the MHC region, HLA typing can serve as a useful initial screening in patients with suspected TAP deficiency.

Two initial reviews on this topic, published in 2000 and 2005 (22, 23), collectively described 15 patients with confirmed TAP deficiency (five TAP1- and ten TAP2-deficient patients). A more recent review, by Hanna and Etzioni in 2014, covered both MHC class I and class II deficiencies (66). Since those earlier publications, additional confirmed cases of TAP deficiency have been identified, some of which have been reported in the literature (28, 30, 31, 32, 33, 36, 39, 40, 41, 42, 43, 48, 50, 52, 67). In the following sections, we discuss the latest insights into the clinical spectrum, immunological characteristics, and treatment options for patients with TAP1 and TAP2 deficiencies. We also comment on patients with deficiencies in β_2_m and tapasin in this regard.

## Clinical spectrum of TAP1 and TAP2 deficiencies

**Figure 3.**
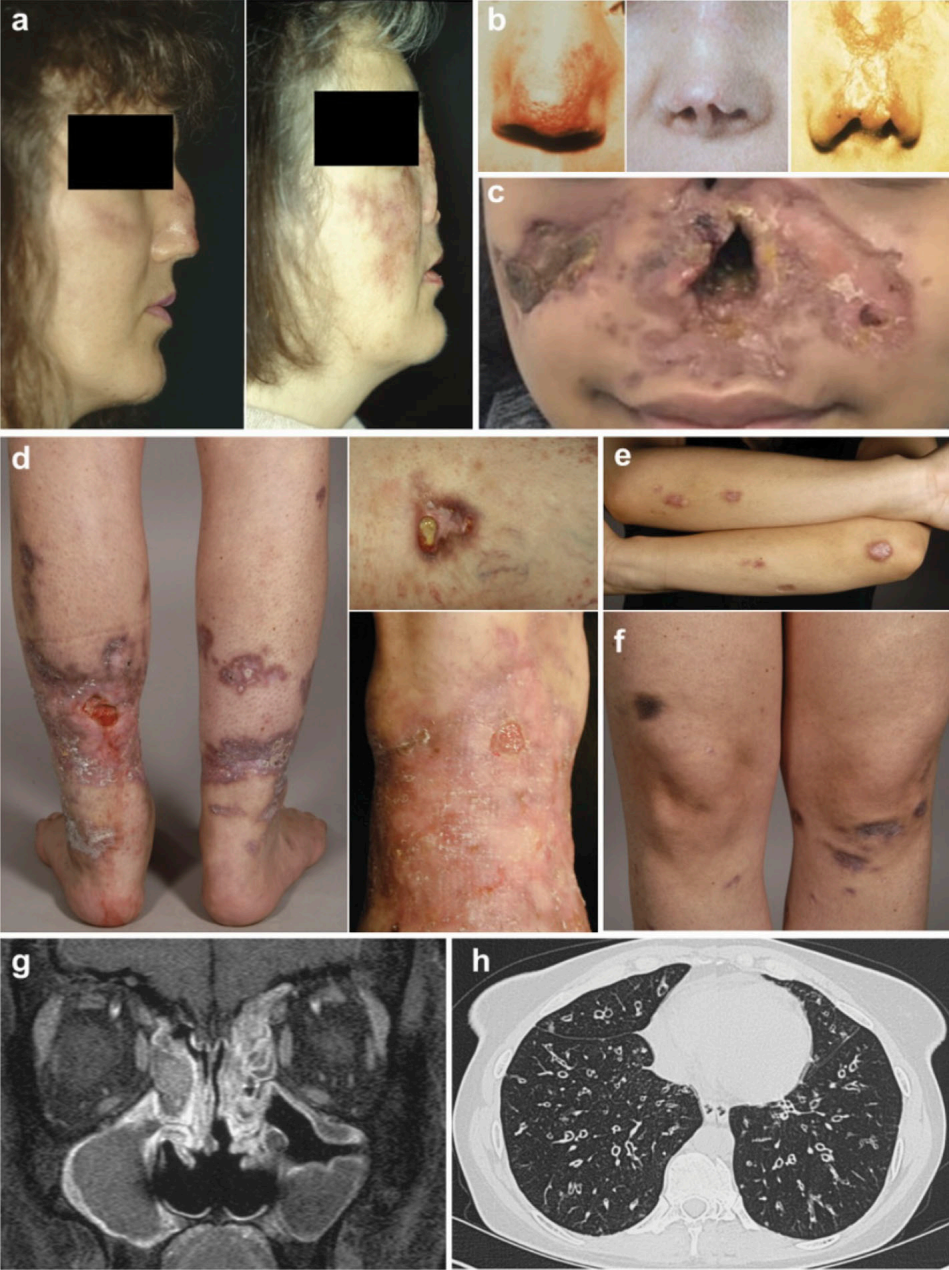
**Clinical manifestations of human TAP deficiency. (a–h)** Clinical manifestations of TAP deficiency affecting the skin (a–f) and respiratory tract (g and h). (a) Progressive midfacial lesions over six years in a patient being under immunosuppressive therapy for suspected GPA; (b and c) different stages of midfacial destruction; (b) left and right panels show the same patient; (c) front view of a patient with severe destruction of the external nose and deep, necrotizing skin lesions extending to the cheeks and philtrum; early lesions such as those shown in b resemble lupus pernio or herpes simplex lesions. The histopathology of these lesions resembles GPA, but GPA does not lead to destruction of the outer nose; (d) typical granulomatous lesions on the lower extremities with pyoderma gangrenosum–like lesions. Leukocytoclastic vasculitis is observed in the same region as the pyoderma gangrenosum–like lesion in the mid-upper panel; (e) scattered erythematous, nodular lesions on the elbows and lower arms. These lesions have been described in different patients as necrobiosis lipoidica, granuloma annulare, or erythema induratum Bazin; (f) hyperpigmented lesions, including one on the right thigh that developed after a blunt trauma; (g) MRI showing chronic maxillary and ethmoidal sinusitis in a patient with pansinusitis (frontal and sphenoidal sinusitis not shown). Chronic sinusitis is a typical early manifestation of TAP deficiency; (h) bilateral bronchiectasis, which typically develops after onset of recurrent pneumonia or chronic spastic bronchitis. These characteristic findings exemplify clinical TAP deficiency, the most common form of bare lymphocyte syndrome type 1.

Clinical manifestations in most TAP-deficient patients include chronic respiratory tract infections and chronic necrotizing (or sometimes non-necrotizing) granulomatous skin lesions (Fig. 3). This unusual combination of symptoms can lead clinicians unfamiliar with TAP deficiency to suspect autoinflammatory conditions, such as granulomatosis with polyangiitis (GPA, formerly Wegener’s granulomatosis), potentially with deleterious consequences for the patients. Below, we provide a more detailed description of the clinical spectrum associated with TAP deficiencies.

### Respiratory tract infections

Respiratory tract infections in TAP-deficient patients typically begin in early childhood, initially affecting the upper respiratory tract (e.g., as chronic sinusitis, otitis media, and rhinitis), often accompanied by nasal polyps and perforation of the nasal septum. Necrotizing granulomatous lesions are frequently found in paranasal sinuses, but rarely in the lower respiratory tract (22). Over time, chronic spastic bronchitis may develop, particularly in children who have undergone sinus surgery for chronic sinusitis. The next stage of disease progression often involves recurrent bacterial pneumonia (caused by *Haemophilus influenzae*, *Streptococcus pneumoniae*, *Staphylococcus aureus*, or *Klebsiella* species), typically associated with the spread of infection from the upper to the lower respiratory tract. If not optimally treated, bilateral bronchiectasis may ensue, with subsequent colonization of the lower airways by *Pseudomonas* and *Streptomonas* species, and sometimes also *Escherichia coli.* Chronic respiratory insufficiency and multidrug-resistant pneumonia are the major complications and causes of mortality in TAP deficiency. However, it is important to emphasize that this fatal trajectory is not a *sine qua non* for patients with TAP deficiency. As will be discussed in more detail below, immunosuppressive drugs, including corticosteroids, should be avoided as soon as the diagnosis is suspected. Additionally, sinus surgeries should be avoided in these patients, as they have not shown any benefit and have instead been associated with disease progression (22).

### Granulomatous skin lesions

Most patients eventually develop granulomatous skin lesions, often in the midface or on the extremities (Fig. 3). While these lesions typically appear years after the onset of respiratory symptoms, sometimes not until adulthood, they have been documented as early as age three in one patient, preceding chronic respiratory infections by several years (68). In another patient, however, granulomatous skin lesions developed as late as at the age 46, here with no previous history of frequent infections (34). The skin lesions typically begin as erythematous macules or papules and progress to nodules that may ulcerate, causing progressive tissue destruction, or heal spontaneously within months, leaving hyperpigmented areas. On the extremities, they may resemble other known dermatoses, such as granuloma annulare, erythema induratum of Bazin, necrotizing sarcoid lesions, or pyoderma gangraenosum. Midface lesions, which carry a high psychological burden, can mimic lethal midline granuloma. Unlike GPA (Wegener’s granulomatosis), these lesions ulcerate the nasal skin and deeper tissues, sometimes leading to the complete destruction of the nose (Fig. 3). Histologically, they originate in the dermis and hypodermis and can progress from a lymphocytic and histiocytic infiltrate to necrotizing granulomatous lesions with vessel infiltration and thrombotic occlusion (unpublished data).

### Potential role of rubella virus in granulomas

Recent evidence suggests that rubella virus, both wild‐type and vaccine strains, may be implicated in granuloma formation in immunocompetent and immunodeficient individuals, including a recent case of TAP1 deficiency (43, 69). Such involvement is particularly relevant in immunodeficiency settings, where T cell or antigen presentation defects may enable viral persistence. Given that granulomas represent a key clinical feature of MHC class I deficiencies, clinicians should consider rubella virus a potential etiological factor. Testing (e.g., via PCR or immunohistochemistry) of granulomatous tissue for rubella can be one part of the diagnostic evaluation.

### Partial phenotypes and HLA-B*07:01–restricted responses

Notably, TAP deficiency has also been identified in asymptomatic individuals. In this context, de la Salle and collaborators described two adult siblings with a homozygous TAP2 mutation (34). HLA class I cell surface expression in these individuals was strongly reduced, but notably three times higher than on other described TAP-deficient patients. These individuals also exhibited nearly normal numbers of CD8^+^ T cells. In one of them, an anti-Epstein–Barr virus (EBV) directed T cell response, mediated by HLA-B*07:01–restricted CD8^+^ T lymphocytes, was documented (34). The anti-EBV reactivity recapitulated earlier findings on anti-EBV reactivity characterized in another TAP-deficient patient (70). In this context, it is worth noting that HLA-B*07:02, like other HLA class I alleles such as HLA-A*02:01, can present TAP-independent peptides (e.g., from signal sequences or from other sources) and by this means elicit CD8^+^ T cell responses (71, 72, 73, 74). This shows that TAP deficiency can remain asymptomatic for several decades, indicating that the occurrence and severity of infection-related (or other related) complications in TAP deficiency may depend not only on the specific genetic defect and environmental factors but also, partially, on the specific HLA class I alleles expressed by the patients.

### Additional clinical manifestations

In addition to these primary features, TAP-deficient patients may experience a wide range of other clinical complications. Chronic encephalomyelitis, cerebral abscesses, mastoiditis, periodontitis, severe dental caries, herpetic keratitis, and ocular toxoplasmosis have been observed, particularly during immunosuppressive treatment initiated under the mistaken assumption of an autoinflammatory disorder (unpublished data). Other presentations, such as colitis, nonerosive polyarthritis, retinal vasculitis, ulcerating laryngitis, pericarditis, and leukocytoclastic vasculitis, have been observed in patients with either long-standing severe airway disease and/or in those previously treated with immunosuppressive drugs for suspected GPA (22, 27) (unpublished data). Table 1 summarizes the wide array of clinical features reported in TAP-deficient patients, detailing their onset, the sequence of symptom evolution, and the patients’ HLA types (to the best of available information), specific mutations in *TAP1* and *TAP2* genes (to the best of available information). The table also includes other genetic alterations, including tapasin and β_2_m deficiencies affecting MHC class I expression.

**Table 1.** Clinical manifestations and genetic features of TAP, tapasin, and β_2_m deficiency

Authors, year (reference)	Age at start of symptom, gender (f, m)		Manifestations (in chronological order, where data available)	HLA type^a^	Genes affected and type of mutation^a^	Country of origin
*Patients with tap deficiency*						
Moins-Teisserenc et al. (1999) (27) ^b^	Age 3 (f)	Chronic rhinitis, otitis, sinusitis; from age 11, bronchial infections with pneumonia and bronchiectasis; from age 27, necrotizing granulomatous skin lesions on nose and legs, progression to nasal destruction under immunosuppression; later developed leukocytoclastic vasculitis, nonerosive polyarthritis, retinal vasculitis, and episodic colitis (on one occasion clostridia toxin positive); at age 36, death from pneumonia		Homozygous HLA-A*11, B*15:02, Cw*08:01, DRB1*15, DQB1*06:01	*TAP2* c.1084delA; p.Ser362Valfs*	Turkey
	Age <5 (f) (aunt)	Recurrent rhinitis and sinusitis since childhood; nasal septal perforation and mild granulomatous lesions on nasal tip; later developed severe necrotizing granulomatous lesions on lower extremities		Same homozygous haplotype		
Caversaccio et al. (2008) (28) Moins-Teisserenc et al. (1999) (27) ^b^	Age 4 (f)	Purulent rhinosinusitis, nasal septal perforation, recurrent middle ear effusion, tympanosclerosis, pharyngitis, mastoiditis, bronchitis, and nasal polyps; from age 10, recurrent pneumonias, bronchiectasis; from age 16: necrotizing granulomatous skin lesions on lower extremities, later involving elbows and forearms		Homozygous HLA-A*26:01, B*49:01, C*07:01, DRB1*13:02, DRB3*03:01, DQB1*06:0, DPB1*15:01	*TAP1* c.819delC; p.Ser274Valfs*	Italy. Male patient described in Plebani et al. (29) shared an identical haplotype
Moins-Teisserenc et al. (1999) (27) ^b^	Age <6 (f) (sister)	Recurrent respiratory infections with bronchiectasis; developed first skin lesions at age 22		Same homozygous haplotype as older sister		
Alemayehu et al. (2023) (30)	Age 3 (m)	From age 3, recurrent pneumonia, gastroenteritis, and tonsillopharyngitis; during infections, developed non-painful skin-colored nodules on extensor limbs		Not stated in article	*TAP2* c.373del; p.Gln125Argfs*8	Ethiopia
Darazam et al. (2023) (31)	Age 26 (f) Age 26–30 (f) (cousin)	Necrotizing granulomatous skin lesions on right foot, later involvement of nose and midface Necrotizing granulomatous lesions on lower limbs with nodules around the nose		Homozygous (details not known)	*TAP2* c.983delC; p.Ala328Glyfs*52	Iran. Three family members with homozygous TAP2 mutations and only minor aphthous ulcers
Espana et al. (2010) (32)	Age 9 (f)	Necrotizing granulomatous skin lesions on both legs; from late childhood, recurrent sinobronchial infections leading to bronchiectasis; at age 39, developed aggressive squamous cell carcinoma within lesional skin; patient died of metastasizing squamous cell carcinoma		Homozygous HLA-A*03:01, Cw*17:01, B*50:01, DRB1*03:01, DQA*05:01/DQB1*02:01, DPB1*04:01	*TAP2* c.628C>T	Spain
Konstantinou et al. (2013) (33)	Age 5 (m)	Recurrent upper and lower respiratory infections; diagnosed with bronchiectasis at age 16; recurrent otitis causing partial bilateral deafness; from age 11, chronic necrotizing granulomatous skin lesions on right lower leg		Not stated in article	*TAP2* c.1345C>T; p.Arg449*	Greece
de la Salle et al. (2002) (34)	Age 43 (m)	Chronic granulomatous erythematous and brownish confluent lupoid papules and plaques on one leg; lesion resolved spontaneously 9 mo after completing antituberculosis treatment A 30-year-old sister carried the same homozygous mutation but had no symptoms at time of analysis. Note: Healthy TAP-deficient individuals reported by Markel shared identical HLA haplotype (35)		Homozygous HLA-A*03:01:01, B*07:02:01, Cw*07:02, DRB1*15, DQB1*06	*TAP2* c.1638+1G>A; p.Gly545Alafs*	Lebanon
Dogu et al. (2006) (36)	Age 6 mo	Meningitis at 6 mo; from age 13, scar-forming necrotizing granulomatous lesions in midface, nose, and philtrum; eye surgery at age 13; toxoplasma pneumonitis at age 14; persistently low IgG, IgA, and B cells		Homozygous HLA-A*26, B*38, DRB1*03, DQB1*02	*TAP1* c.1312C>T; p.*	Turkey
	Age 17 (f)	Recurrent lower airways infections with subsequent bronchiectasis		Same homozygous haplotype		
Donato et al. (1995) (37)	Age 4 (f) Age 7 (m)	Recurrent sinobronchial infections, severe bilateral nasal polyposis, and bronchiectasis Recurrent pulmonary infections progressing to bronchiectasis; also had nasal obstruction, pansinusitis, and chronic otitis media		Homozygous HLA-A3, B63, DR4, DR53, DQ3	*TAP2* c.757C>T; p.Arg253*	Morocco
Gao et al. (2016) (38)	Age 4 (f)	Recurrent pneumonia with bronchiectasis; from age 10, ulcerating skin lesions on elbows with human herpes virus (HHV) and EBV positivity; marked improvement following HSCT		Homozygous HLA-A*24:02, B*35:02, Cw*04:01, DRB*03:01, DQB*02:01, DPB*05:01	*TAP1* Homozygous deletion spanning TAP1 exon 3 to exon 11	Pakistan
Hanalioglu et al. (2017) (39)	Age 4 (f)	Sino bronchial infections resulting in bronchiectasis (age at first symptoms not reported)		Homozygous HLA-A*26, B*38, C*12, DRB1*03, DQB1*03	*TAP1* c.2104_2105insC; p.Gln702Profs*27	Turkey
Villa-Forte et al. (2008) (40)	6 mo	Recurrent pneumonia with bronchiectasis by age 6; from age 12, multiple ulcerating granulomatous skin lesions on both legs, initially resolving, later chronic; progressive disease under high-dose glucocorticoids, cyclophosphamide, methotrexate, azathioprine, and infliximab for suspected GPA		Homozygous HLA-A*01, B*08, Cw*07, DRB1*03, DRB3, DQB1*02	*TAP1* c.2239G>A; p.*	Country not stated (Brazilian origin)
Law-Ping-Man et al. (2018) (41)	Age 4	Ulcerating granulomatous skin lesions on left cheek, gluteal region, and limbs; at age 11, spastic bronchitis with bronchiectasis		Homozygous HLA-A*02:01, B*44:02, C*05:01, DRB1*04:01,DQB1*03:01, DPB1*04:01	*TAP1* c.1879A>T; p.Lys627*	Country not stated (Caucasian origin)
Moins-Teisserenc et al. (1999) (27) *	Age 12 (f)	Chronic sinusitis and bronchitis since childhood, later bronchiectasis; from age 35, granulomatous skin lesions on legs and midface with nasal destruction; at age 39, cerebral abscesses under immunosuppression; at age 46, hypopharyngeal ulceration; at age 47, leukocytoclastic vasculitis		Homozygous HLA-A*03, B*15:01, Cw*03, DR*13:01, DQ*06:03	*TAP1* Deficiency demonstrated by lack of TAP1 protein. No TAP1 sequencing data published	Belgium
Moins-Teisserenc et al. (1999) (27) *	Age 3 (f)	Initial presentation with necrotizing granulomatous lesions on both legs, later involving nose and midface; chronic sinusitis diagnosed at age 27		Homozygous HLA-A*23:01, B*49:01, Cw*07:01, DRB1*03:01, DQB1*02:01	*TAP1* Deletion due to frameshift mutation with premature termination	Belgium
Parissiadis et al. (2005) (42)	Age 14 (f)	Unilateral ocular toxoplasmosis with chorioretinitis and profound loss of vision Older brother with spastic bronchitis and chronic bacterial colonization of the lower airways		Homozygous HLA-A*24, B*14, Cw*08, DRB1*13, and DQB1*06	*TAP1* c.1564C>T; p.Arg522*	Country not stated (French origin)
Wang et al. (2024) (43)	Age 27 (f)	Non-painful plaques on right leg; biopsy showed suppurative granulomatous inflammation with caseation; rubella virus–induced granulomatous disease diagnosed via metagenomic sequencing		Not stated in article	*TAP1* c.1151C>G; p.Ser384*	China
Maeda et al. (1985) (44) Watanabe et al. (1987) (45) de la Salle et al. (1999) (46) Furukawa et al. (1999) (47)	Age 15 (f)	Rhinitis with nasal polys, followed by sinusitis and panbronchiolitis; at age 28, granulomatous skin lesion on the left leg		Homozygous HLA-A*24:02, B*40:06, C*15, DRB1*08:03, DQB1*06:01, DPB1*05:01	*TAP1* c.778+1G>A	Japan
Plebani et al. (1996) (29) de la Salle et al. (1999) (46)	Infancy (m) Not stated (m)	Sinobronchial infections from infancy, progressing to bronchiectasis; from age 8, deep skin ulcers on extremities; died at age 23 from respiratory failure Older brother died at age 20 of a cerebral abscess, with bronchiectasis and thymic atrophy at autopsy		Homozygous HLA-A*26:01, B*49:01, C*07, DRB1*13:02, DRB3*03:01, DQB1*06:04, DPB1*15:01	*TAP1* c.819delC; p.Ser274Valfs*	Italy. Shares identical haplotype with Italian patients described in (27) and (28)
Gadola et al., unpublished data	<Age 6 (f)	Upper respiratory tract infections and herpetic fever blisters, herpetic keratitis, acute hearing loss, and generalized exanthema following smallpox vaccination; from age 13, granulomatous skin lesions on legs; later (adulthood) recurrent strokes and progressive atactic syndrome under immune suppression		Homozygous HLA-A32, B57	*TAP2* c.711+1G>C	Poland
Bhattarai et al., 2025 (48)	Age 7 (f)	Recurrent fever and respiratory infections; severe varicella-zoster and herpes simplex virus infections; pneumonia with disseminated vesicular rash; persistent poorly healing skin lesions, pyoderma gangrenosum-like cutaneous ulcers, and vasculitic rash		Homozygous	*TAP1* Not known	Nepal. Treated with IgRT and antimicrobial prophylaxis
Ramalingam et al. (2024) (49)	Age 3 (m)	Recurrent pneumonia with good clinical response to HSCT		Not stated in article	*TAP2* c.1733C>T; p.Ala578Val	India
Samarkandy et al. (2024) (50)	Age <10	Recurrent otitis media with effusion before age 10; from age 10, granulomatous plaque-like lesions on both legs; partial response to glucocorticoids and thalidomide, followed by severe relapse after thalidomide withdrawal		Not stated in article	*TAP1* and *TAP2* Homozygous 17 kb deletion spanning TAP1 exon 8 to TAP2 exon 7	Saudi Arabia
*Patients with tapasin deficiency*						
Yabe et al. (2002) (51)	Age 44 (f)	Chronic glomerulonephritis for 10 years; later developed herpes zoster and gastric and colonic polyps		Homozygous HLA A*26:01, B*15:01, Cw*03:03, DRB1*15:01	*TAPBP* Homozygous deletion spanning introns 3–7	Japan
Elsayed et al. (2024) (52)	Age 24 (m)	Recurrent bronchitis, sinusitis, and otitis media; diagnosed with bronchiectasis at age 24; at age 39, herpes zoster and postherpetic neuralgia at thoracic dermatomes		Not stated in article	*TAPBP* c.312del; p.Lys104Asnfs*6	Turkey
Ramalingam et al. (2024) (49)	Age 9 (m)	Recurrent respiratory infections with wheezing and hypoxia; at age 10, CT showed bilateral diffuse fibrosis and bronchiectasis; died at 4 mo post-HSCT due to severe viral and bacterial infections and poor lymphocyte reconstitution (2%–5%)		Not stated in article	*TAPBP* c.312del; p.Lys104Asnfs*6	India
*Patients with β*_*2*_ *m deficiency*						
Waldmann et al. (1990) (53) Wani et al. (2006) (54)	Age 21 (f)	Necrobiosis lipoidica diabeticorum on legs after miscarriage; low IgG and albumin; skeletal anomalities (short bowed limbs). At age 25, developed idiopathic thrombocytopenic purpura; 7 mo after splenectomy, died from pneumonia, thrombocytopenia-related hemorrhage, and septic shock. Sibling: Low IgG and bone anomalies, but asymptomatic at follow-up; anomalities attributed to FcRn deficiency		Not stated in article	*β*_*2*_ *m* Homozygous mutation in signal peptide region; residual MHC class I expression	Country not stated
Ardeniz et al. (2015) (55)	Age 9 (f)	Subcutaneous nodules during flu-like infection; treated empirically for suspected tuberculosis; at age 12, left lung abscess; in adulthood, nasal perforation and ulcerated violaceous skin lesions on all extremities; bronchiectasis confirmed by CT; severe hypoproteinemia at age 31. Brother: Bronchiectasis and low serum proteins; three siblings died young, one with chronic granulomatous skin lesions		HLA A*11/A*24, B*27/B*40, C*15/C*15, DRB1*04/07	*β*_*2*_ *m* Homozygous intron 1 mutation causing aberrant splicing, frameshift, and premature stop in exon 2; no detectable β2m or MHC class I expression	Turkey

a To the best of our available information.

b The clinical details are based on the personal records of S.D. Gadola, who identified and followed these patients over time. Due to word count restrictions, only a summarized version of these cases was included in the original Lancet publication.

### Viral infections

Although MHC class I molecules are critical for CD8^+^ T cell development as well as for presentation of viral antigens to CD8^+^ T cells, severe viral infections are generally not a hallmark of TAP deficiency. Nonetheless, one patient developed acute left-sided hearing loss and a transient generalized exanthema days after receiving a smallpox vaccine (a live, non-attenuated vaccine) while tolerating other childhood immunizations (unpublished data). This patient and her sister also experienced recurrent severe herpetic fever blisters, and after immunosuppressive treatment for skin lesions, they both developed herpetic eye inflammation (confirmed by PCR in the sister), cerebral complications including cerebral atrophy, and recurrent strokes possibly because of herpetic cerebral vasculitis (unpublished data). More recently, a 7-year-old Nepalese girl with TAP1 deficiency presented with pneumonia, disseminated vesicular rash, and persistent lesions caused by varicella-zoster and herpes simplex viruses, requiring prolonged antiviral treatment (48). Thus, TAP-deficient individuals may be at risk for severe viral infections, a risk that can be amplified by immunosuppressive treatments.

### Cancer risk

MHC class I–mediated presentation of tumor-specific antigens to CD8^+^ T cells is a key mechanism of immunosurveillance against malignancies. However, few patients with long-standing TAP deficiency have been diagnosed with cancer. Of these, we are aware of three patients, who developed squamous cell carcinoma arising from persistent granulomatous skin lesions (32, 35) (unpublished data). Two of these three died from their disease. Another patient was diagnosed with a nonspecified cancer, underwent chemotherapy, and subsequently died of sepsis (68) (Willemsen, R., personal communication).

## Immunopathological aspects of TAP deficiency

Several patients with TAP deficiency exhibit reduced frequencies of peripheral CD8^+^ T cells, likely due to impaired positive selection of native CD8^+^ T cells in the thymus, a process dependent on MHC class I molecules (see Introduction). However, some patients have been shown to display normal or even elevated CD8^+^ T cell levels. In some cases, this increase has been attributed to progressive accumulation of CD8^+^ γδT cells, whereas in others, it has reflected expansions of CD8^+^ αβT cells. It is not unlikely that a subset of the latter might include mucosa-associated invariant T (MAIT) cells and/or CD1-restricted T cells. Notably, γδT cells expressing the T cell receptor Vδ1 chain, normally a rare subset in healthy individuals, are significantly expanded in the peripheral blood of many TAP-deficient patients, resulting in an inversion of the typical Vδ2 to Vδ1 ratio (22, 27).

The number of NK cells in peripheral blood is generally normal in cases of TAP deficiency, although increases have been observed during infections in some cases (13, 75, 76). In normal conditions, NK cell responses to human cytomegalovirus (HCMV) often involve expansions of NKG2C+ NK cells, which are typically shaped by interactions with HLA-E known to bind N-terminal leader (signal) sequences of classical MHC class I heavy chains (HLA-A, -B, or -C) (77, 78, 79). Such expansions have also been observed in TAP-deficient patients (77). Furthermore, NK cells have been shown to exhibit a polyclonal killer cell inhibitory receptor profile, express high levels of CEACAM1, and retain at least partial functional responses in TAP-deficient patients (80, 81, 82). Upon in vitro activation, NK cells have been reported to efficiently kill autologous B lymphoblastoid cells and skin fibroblasts (27, 75, 83), but not autologous phytohemagglutinin T cell blasts (81).

Aside from a single study (31), deep immunophenotyping has not been extensively performed in TAP-deficient patients. In the aforementioned study, mass cytometry analysis of peripheral blood mononuclear cells (PBMCs) from two TAP2-deficient individuals revealed largely normal myeloid and B cell counts, apart from a slight plasma cell increase. NK cell numbers were elevated, with higher frequency of CD56^dim^NKG2C^+^ cells suggestive of prior HCMV infection. T cell analysis showed overall normal γδ and αβ T cell counts, with CD4 T cells (including T helper and regulatory T subsets) within normal limits. In contrast, CD8^+^ naïve T cells were markedly reduced, though increased effector memory cells maintained total CD8 counts, resulting in an abnormally high CD4/CD8 ratio among naïve T cells. Additionally, both MAIT and invariant NK T cell numbers were elevated. Taken together, these findings indicate a relatively mild impact of TAP2 deficiency on T lymphocyte differentiation, characterized by a decrease in CD8^+^ naïve T cell thymus output and increased invariant T cells (31).

The pathophysiology of granulomatous skin lesions in TAP deficiency may involve NK and Vδ1 T cells that, when activated during infections, may not be properly regulated. Supporting this view, dense infiltrates of NK and γδT cells have been documented in granulomatous skin lesions of TAP-deficient patients (27). Similarly, CD4^+^ and CD8^+^ T cell infiltrations in granulomatous lesions of β_2_m deficiency comprised mainly of γδT cells (55). These lesions also show high expression of MHC class II on infiltrating cells, indicating cellular activation as well as high expression of Ig-like transcript 2, an MHC class I–binding inhibitory receptor on lymphoid and myeloid cells (27).

## Treatment options in TAP deficiency

The guiding principle for managing TAP-deficient patients should be “*primum non nocere*” (first, do no harm). This said, chronic granulomatous skin lesions or chronic spastic bronchitis may tempt clinicians to initiate immunosuppressive drug therapies, commonly starting with prednisone. While initial short-term responses can be encouraging, extensive patient histories indicate that any form of immunosuppression ultimately provides no benefit and can cause significant harm in TAP-deficient individuals (22, 27) (unpublished data). Below, we provide a more detailed description of clinical TAP deficiencies.

### Antibiotics and supportive care

The judicious use of antibiotics, saline nasal washings, and chest physiotherapy is vital for long-term management. In early disease stages, before bronchiectasis is established, antibiotics, such as doxycycline (known for favorable tissue pharmacokinetics, anti-inflammatory properties, and relatively low propensity for resistance), may be used. Also, some patients seem to have benefitted from long-term prophylaxis with sulfamethoxazole/trimethoprim against *Toxoplasma* infections, combined with regular intravenous immunoglobulins (36). For established bronchiectasis with *Pseudomonas* or *Streptomonas* airway colonization, inhaled antibiotics commonly employed in cystic fibrosis (colistin, tobramycin, aztreonam lysine, and levofloxacin) can be considered. Notably, intermittent inhaled colistin has maintained stable disease over several years in one TAP-deficient patient (unpublished data).

### Antituberculosis therapy and related observations

Several patients have also received quadruple tuberculostatic therapy for suspected tuberculosis, despite no evidence of *Mycobacterium tuberculosis* (22). Interestingly, some patients improved during this therapy, and, in at least two cases, skin lesions healed (unpublished data). In one instance, a patient treated with rifampicin and pyrazinamide experienced the reappearance of skin lesions upon discontinuation of rifampicin. The lesions regressed again when rifampicin was reinstated after discontinuing pyrazinamide (unpublished data). Rifampicin has a broad antibacterial spectrum, while pyrazinamide is specific for *M. tuberculosis*. In another patient, stable control of lung infections correlated with the healing of skin lesions (unpublished data). Conversely, one patient originally reported by Law-Ping-Man et al. (41) experienced worsening skin lesions in association with intermittent pulmonary infections (Adamski, H., personal communication). These cases suggest that bacterial lung infections may trigger or reactivate NK and γδT cells in skin lesions. Notably, in this context, the patient described above, treated with long-term sulfamethoxazole/trimethoprim and immunoglobulins, developed severe skin lesions during therapy (Dogu, F., personal communication).

### Immunomodulatory approaches and cautionary tales

One patient, that we are aware of, received the TNF-α inhibitor infliximab. While the cutaneous lesions seemed to improve temporarily, the patient later developed aggressive metastatic squamous cell carcinoma on a chronic skin ulcer, ultimately leading to death at the age of 54 (35) (unpublished data). Therefore, we do not recommend such treatments, as the long-term risks may outweigh any short-term benefits. Immunomodulatory treatment with IFN-α or IFN-γ in TAP-deficient patients have been associated with worsening skin lesions and systemic side effects, such as severe fatigue and malaise (22). However, in the rare cases of tapasin deficiency, IFN-α may be a viable therapeutic approach. Type I IFNs can upregulate the MHC class I–loading complex molecules and by this means enhance MHC class I expression on the surface of PBMCs from tapasin-deficient patients (52).

### Definitive treatment options

With respect to curative treatment, gene therapy represents a theoretical option for restoring MHC class I expression in all nucleated cells, although it is not yet (and far from) clinically available. Hematopoietic stem cell transplantation (HSCT) is a possible option but has notable limitations in that MHC class I expression will only be corrected in donor-derived hematopoietic cells. Nevertheless, HSCT has been performed in two patients, as reported by Gao et al. (38) and Tsilifis et al. (67). In the first case, a patient with combined TAP1 and TAP2 deficiencies underwent low-intensity conditioning followed by HSCT at age 13 (38). 15 years later, this patient remains free of infections, has had a normal first pregnancy, and shows donor-derived hematopoietic cells with normal HLA class I expression and a typical CD8^+^ T cell fraction (67). By contrast, the second patient, an 11-year-old with TAP1 deficiency, died ∼2 mo after transplantation due to severe graft-versus-host disease and concurrent pulmonary infections with cytomegalovirus (CMV) and parainfluenza virus type II (67).

## Other human immune deficiencies affecting MHC class I expression

An individual with a large deletion encompassing both the *TAP1* and *TAP2* genes as well as the proteasome-related proteasome subunit beta type-8 gene was reported by Gao et al. (38) and has been described above. In addition to TAP mutations, a few other documented genetic defects can lead to decreased HLA class I expression (84). The most severe cases involve simultaneous loss of HLA class I and II expression, also known as bare lymphocyte syndrome type 3 (85). The latter representing a very rare condition resulting in severe combined immunodeficiency with life-threatening infections by bacteria, viruses, fungi, and other opportunistic organisms presenting in early childhood.

### β_2_m deficiency

Mutations in *β*_*2*_*m* genes have been described in two brother-sister pairs (53, 55). Both female patients developed granulomatous dermatitis and pulmonary infections mirroring those seen in TAP-deficient patients. One female patient displayed severe disease with disfiguring midfacial and limb granulomas, herpetic blisters on the soles, CMV retinitis, and ultimately died from toxoplasmosis (55). The other female patient underwent splenectomy due to idiopathic thrombocytopenia and died of bilateral pneumonia and septic shock at age of 40 (53). In contrast, neither of the male siblings showed notable immune deficiencies into their twenties, at which point they were lost to follow-up (53, 55). Notably, β_2_m-deficient patients exhibit distinct biochemical and immunological phenotypes compared to those with TAP deficiency (55). These differences are due to the pleiotropic functions of β_2_m as a scaffolding protein for both classical and nonclassical MHC class I molecules, including CD1 proteins (CD1a-d), MR1, hemochromatosis protein (HFE), and the neonatal Fc receptor (FcRn) (54). The FcRn binds albumin and IgG, extending their catabolic half-lives, which explains why all β_2_m-deficient patients exhibited severe hypoalbuminemia and very low IgG serum levels (53, 55). While MR1 and HFE expression were not analyzed, CD1a-c expression was markedly reduced in one of the female and in the male patients described above (55). Interestingly, CD1d expression remained normal, consistent with published observations that this isoform can be expressed independently of β_2_m in human cells (86). On a cellular level, the patients demonstrated a highly biased TCR Vβ repertoire among CD8^+^ αβ T cells, low B cell counts, and strikingly increased CD8^+^ γδT cells, which were more than six times the upper normal limit (55).

### Tapasin deficiency

To date, three cases of inherited deficiency of tapasin have been reported (49, 51, 52). The first involved is an adult female with chronic glomerulonephritis. Her tapasin defect was discovered during a pre-kidney transplant workup. The patient suffered from digestive polyps and had a history of varicella-zoster virus infection. Of interest, this patient did not exhibit chronic respiratory or cutaneous symptoms as typically seen in TAP deficiency (51). The second (52) and third cases (49) have been more recently described. These two patients presented with bronchiectasis and recurrent respiratory tract infections that largely resembled the clinical spectrum of TAP deficiencies (52).

### Additional reports

A few asymptomatic individuals with modest reductions in HLA class I expression have been reported (34, 87). They were heterozygous for HLA class I and II, with consanguineous parents. In these cases, HLA-class I expression was inducible by inflammatory cytokines, suggesting a possible transcriptional rather than a structural defect. Finally, other patients with confirmed low MHC class I expression and clinical manifestations resembling TAP deficiency have been observed, but underlying molecular defects have not yet been described (68, 88) (unpublished data).

## Conclusion

Inborn defects in the MHC class I pathway cause a wide range of clinical manifestations, ranging from chronic infections, primarily affecting the respiratory tract, to autoinflammatory granulomatous skin lesions driven by innate immune cells, such as NK and Vδ1 γδT cells. These granulomatous lesions, along secondary immune complex–mediated leukocytoclastic skin vasculitis, can occasionally be misdiagnosed as GPA (formerly known as Wegener’s disease) or other autoimmune conditions. Treatment should tightly be focused on infection control and prophylaxis. Immunosuppressive therapy is contraindicated. Such therapy can result in severe infectious complications and ultimately fatal lung damage. Recent long-term follow-up data have revealed new insights into TAP-deficient patients, including the development of skin cancer in later stages of the disease, possibly arising from the chronic granulomatous skin lesions. Additionally, severe herpesvirus infections have been observed, contradicting the prevailing belief that viral attacks have minimal consequences. Finally, some individuals with significant reduction in MHC class I expression present with minimal or no clinical symptoms, highlighting the heterogenous nature of this disorder. From a diagnostic standpoint, HLA typing in suspected cases can help identify homozygosity within the MHC region or shared haplotypes that may harbor *TAP* (or *TAPBP*) gene mutations. Next-generation sequencing offers an even more precise method to confirm the presence of pathogenic variants in *TAP* genes, facilitating earlier and more accurate diagnoses. Looking ahead, emerging gene therapy modalities may hold promise for correcting these severe immunodeficiencies in the future. In conclusion, advancing our understanding of the genetic mutations and molecular mechanisms underlying MHC class I deficiency will be crucial in identifying predictive markers for disease progression, guiding individualized treatment strategies, and optimizing patient outcomes. Long-term surveillance and follow-up of affected patients will also remain essential for uncovering late-onset complications and guiding ongoing clinical practice and patient management.

## Data Availability

No new data were generated or analyzed in the present review. With respect to patient-specific observations described, underlying data are not publicly available due to patient privacy issues. Further nonconfidential information can be made available from the corresponding author upon reasonable request with the permission of third party.
